# POIS-10 Māori: Outcomes and Experiences in the Decade Following Injury

**DOI:** 10.3390/mps4020037

**Published:** 2021-05-20

**Authors:** Emma H. Wyeth, Sarah Derrett, Vicky Nelson, John Bourke, Sue Crengle, Gabrielle Davie, Helen Harcombe

**Affiliations:** 1 Ngāi Tahu Māori Health Research Unit, Department of Preventive and Social Medicine, Dunedin School of Medicine, University of Otago, P.O. Box 56, Dunedin 9054, New Zealand; v.nelson@otago.ac.nz (V.N.); johnny.bourke@otago.ac.nz (J.B.); sue.crengle@otago.ac.nz (S.C.); 2 Injury Prevention Research Unit, Department of Preventive and Social Medicine, Dunedin School of Medicine, University of Otago, P.O. Box 56, Dunedin 9054, New Zealand; sarah.derrett@otago.ac.nz (S.D.); gabrielle.davie@otago.ac.nz (G.D.); helen.harcombe@otago.ac.nz (H.H.); 3 Burwood Academy Trust, Private Bag 4708, Christchurch 8140, New Zealand; 4 Menzies Health Institute, Griffith University, Brisbane, QLD 4222, Australia

**Keywords:** injury, injury outcomes, Māori health, indigenous health, disability, well-being, flourishing, person-reported outcomes, longitudinal cohort study

## Abstract

Injury-related disability burden extends well beyond two years post-injury, especially for Māori (Indigenous) New Zealanders. Māori also experience greater difficulty accessing health services. This prospective cohort study extension uses mixed-methods and aims to understand and identify factors contributing to long-term experiences and outcomes (positive and negative) at 12 years post-injury for injured Māori and their whānau (families), and explore the barriers and facilitators to whānau flourishing, and access to health and rehabilitation services. Five hundred and sixty-six Māori, who were injured between 2007–2009, participated in the Prospective Outcomes of Injury Study (POIS). Of these, 544 consented to long-term follow up, and will be invited to participate in a POIS-10 Māori interview at 12 years post-injury. We anticipate a 65% follow-up rate (~*n* = 350). Aligned with the Meihana Model, interviews will collect information about multiple inter-related dimensions. Administrative injury and hospitalisation data up to 12 years post-injury will also be collected. Regression models will be developed to examine predictors of long-term health and disability outcomes, after adjusting for a range of confounders. POIS-10 Māori will identify key points in the injury and rehabilitation pathway to inform future interventions to improve post-injury outcomes for Māori and whānau, and will highlight the support required for Māori flourishing post-injury.

## 1. Introduction

Injury is responsible for one-third of disability in New Zealand and is costly [1,2,3]. In the 2018/2019 year alone, New Zealand’s no-fault injury insurer, the Accident Compensation Corporation (ACC), received 2,027,789 injury claims, and spent $4.4 billion supporting injured people [2]. There is accumulating evidence that the injury-related disability burden extends well beyond two years post-injury, and that for some, the need for support increases over time. Of particular concern, Māori (Indigenous New Zealanders) are more likely to experience disability and poor health outcomes in the years following injury compared to non-Māori [1,3,4,5]. Government agencies, such as ACC and the Ministry of Social Development, are working collaboratively to increase understanding of the long-term impacts of injury on well-being outcomes across the life course. However, despite these efforts, Māori experience inequities in access to ACC-funded services, with lower rates of receipt for almost all of the range of services funded, compared to non-Māori [6,7]. Our Māori Prospective Outcomes of Injury Study (POIS-10 Māori), provides a rare opportunity to explore factors that influence disability, well-being and health outcomes for injured Māori in New Zealand, 12 years after their sentinel injury event (SIE), and to identify barriers that Māori face in receiving and accessing ACC and injury-related healthcare.

### 1.1. The Burden of Injury for Māori in New Zealand

Like most other health issues, Indigenous populations experience greater injury burdens than non-indigenous groups in their respective countries [8]. For example, the incidence of severe injuries among Aboriginal Canadians is nearly four times higher than non-Aboriginal Canadians [9]. Sámi males in Sweden [10], Sámi males and females in Finland [11], Roma females in Serbia, and Roma males and females in Bulgaria [12], all have higher injury morbidity and mortality rates than their non-indigenous counterparts [8]. In Australia, the total injury burden for the Aboriginal population is three times higher than for the total Australian population [13]. 

Similarly, Māori in New Zealand experience marked health inequities compared to non-Māori [14,15], including for injury and disability [4,5]. Māori have higher age-adjusted rates of disability than non-Māori (32% vs. 24%, respectively) [1], have higher rates of hospitalisation due to injury (18 per 1000 vs. 11 per 1000, respectively) and twice the mortality rate due to unintentional injuries [4,16]. Furthermore, Māori, compared to non-Māori, are estimated to experience at least twice as much health loss (in disability-adjusted life years) due to injury (12% vs. 6%, respectively) [3]. Despite such inequities, Māori often have lower rates of healthcare access and receive lower-quality care compared to non-Māori (e.g., fewer general practitioner visits, diagnostic tests, medical interventions, poorer outcomes after illness and fewer disability allowances [16,17]).

Ethnic and racial inequities for injury-related disability and health service access are prevalent worldwide. However, very little attention has been paid to quantifying the risks and predictors of injury, and injury outcomes for Indigenous peoples with a view to improving outcomes. In New Zealand, although existing data provide valuable information about the burden of injury for Māori, they are either sourced from cross-sectional data collections, are not self-reported or are crude estimates [6,7]. Consequently, they do not help inform the in-depth understanding of the lived pathways to positive (and negative) outcomes for injured Māori and their whānau that are required to thoroughly address the existing inequities. There is a paucity of research that explores a wide range of injury types and predictors of Māori post-injury outcomes, especially from a Māori-led perspective. Māori injury literature often focuses on specific risk factors for injury (e.g., alcohol consumption [18]) or is limited to specific injury causes (e.g., work-related) and severe injuries (e.g., burns, spinal cord injuries) or only injuries requiring hospitalisation. Outside of our own research, no studies in New Zealand, or overseas, have prospectively and longitudinally collected rich person-level data to investigate Māori or Indigenous post-injury outcomes, or the impacts of injury for whānau.

### 1.2. The Need for POIS-10 Māori: Evidence from our Existing Mixed-Methods Research

Our earlier POIS, was a longitudinal study recruiting 566 Māori (20% of 2856 participants) ACC entitlement claimants who were injured between 2007–2009 [19,20]. ACC entitlement claims are for injuries warranting increased ACC support (e.g., earnings-related compensation, home-help or transportation support) and largely exclude injuries requiring less than one week off work or medical fee-only claims [19]. The POIS collected detailed quantitative information directly from participants about a wide range of pre-injury, injury-related and post-injury characteristics at 3, 12 and 24 months following the original sentinel injury event (SIE) [20,21]. Qualitative interviews were also conducted with 15 Māori POIS participants living in the Otago/Southland region at ~6 and 12 months post-injury. Importantly, POIS Māori participants experienced a wide range of injury types (e.g., fractured upper limb, concussion), causes (e.g., motor vehicle crash, assault) and settings (e.g., work, home). Unlike previous injury cohort studies, POIS combined detailed interview data with linked administrative injury electronic data (e-data) from ACC (e.g., earnings-related wage compensation, healthcare utilisation, treatment costs, and additional injury events) and the Ministry of Health’s National Minimum Data Set (NMDS) of injury-related hospitalisations in the 24-months post-SIE.

Our POIS findings have shown that different pre- and post-injury factors are related to disability and health outcomes for Māori at specific time points post-SIE [20,22,23,24,25,26]. For instance, financial security, injury severity, occupation type and job tasks impact whether injured Māori are working or not three months after injury [23]. Furthermore, having two or more chronic conditions, difficulty accessing healthcare services for injury, being hospitalised and having inadequate household income predict disability at 24 months post-injury [26]. Alarmingly, compared to non-Māori, Māori experience higher levels of adverse outcomes (pain and discomfort, psychological distress, difficulties with mobility and usual activities) than non-Māori at 3 [22], and 12 months post-injury [27]. Māori hospitalised for injury are also 1.8 times more likely to experience disability 24 months post-injury compared to hospitalised non-Māori [25].

Indeed, a considerable proportion of injured Māori POIS participants experienced difficulties 24 months post-injury. For instance, 72% of Māori participants reported at least one of a range of adverse outcomes (disability, lower health-related quality of life (HRQoL), and non-return to paid employment) at 24 months post-SIE, compared to 17% pre-SIE. Various factors such as comorbidities or labour market dynamics may influence these outcomes but, importantly, 46% of Māori also specifically reported ongoing problems from their SIE at this time point. Even when injured Māori *were* able to access health and rehabilitation services, 19% still experienced difficulties 24 months post-injury compared to 9% pre-injury [26]. Given the known burden at 24 months post-injury, it is critical that we investigate long-term outcomes (up to 12 years post-SIE) for injured Māori. Our POIS-10 Māori study will enable us to explore whether such burdens decrease or increase over time, what factors facilitate positive (and negative) outcomes, using Māori frameworks to ensure relevance to injured Māori. To the best of our knowledge, POIS, and by extension POIS-10 Māori, has the largest longitudinal cohort of injured Māori (or other Indigenous) adults, and is one of the largest longitudinal studies of Māori adults. We will follow-up with Māori POIS participants to understand long-term post-SIE outcomes, and their predictors. The qualitative component of POIS-10 Māori will specifically enable further insights to be gained into the barriers and facilitators of accessing healthcare and injury-related services for Māori.

#### 1.2.1. Whānau Flourishing as a Protective Factor to Injury

Despite adverse injury outcomes for Māori post-SIE, most POIS Māori participants still reported satisfaction with life at three months post-injury [28]. It is likely that participants’ whānau act as a protective factor against such negative outcomes. Our previous qualitative work highlights the importance of whānau during injury recovery and rehabilitation processes, and shows that injury impacts extend to whānau, as with other aspects of health [29,30,31,32]. Whānau flourishing has been described as the “capacities of whānau to undertake expected roles and functions” [33] (p. 33). Many factors (both external and internal) can determine an individual’s capacity to function and undertake usual roles, through their impact on physical, mental, spiritual and whānau well-being [33,34]. Whilst there is existing research about the causal factors of languishing for Māori whānau, there is much less research about the key determinants of positive flourishing and most existing research tends to focus on negative aspects, e.g., whānau exclusion, deprivation and disconnectedness [34]. It is of utmost importance to understand and identify the key factors that facilitate whānau flourishing, and ensure that Māori can flourish, even while enduring significant adversity [33,34,35], such as a substantial injury. There is a growing focus upon empowerment, self-management and independence of Māori and whānau, in health and health research [34]. Obtaining well-being and flourishing for an injured Māori individual is dependent upon the collective well-being and flourishing of their whanau [33,34].

#### 1.2.2. A Kaupapa Māori Approach to Understanding Outcomes of Injury

**Figure 1:**
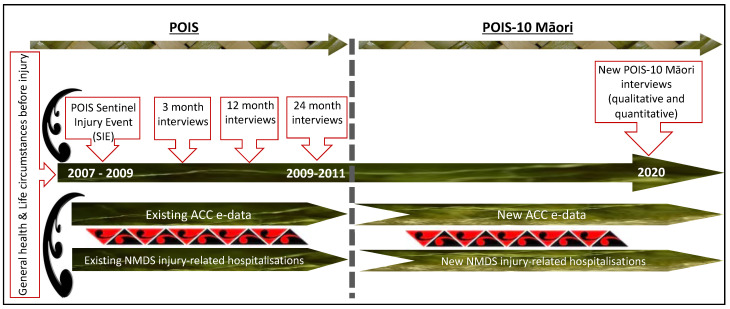
POIS-10 Māori Overview.

This project will be underpinned by kaupapa Māori principles [35], with a non-deficit approach whereby the problem is not ‘being Māori’; instead, rather than locating the causes of inequities and adverse outcomes with the individual, system and structural biases are explicitly investigated [15]. It also aligns with Māori data sovereignty principles [36]. Māori processes and practices will be prioritised in all aspects of the project; it is Māori-led and the majority of the research team and advisors are Māori, importantly, enabling Māori “to have tino rangatiratanga over research that investigates Māori issues” [35] (p. 37). POIS-10 Māori (Figure 1) has been intentionally designed to only include Māori participants so we can deliberately explore and understand the key factors, barriers and facilitators that influence post-injury outcomes of importance to Māori.

**Figure 2:**
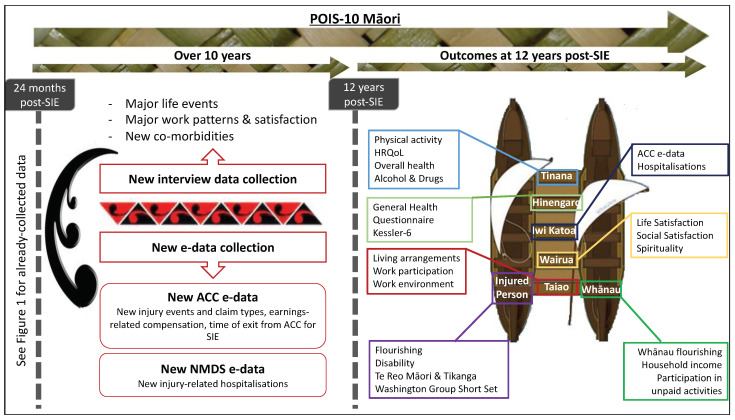
New POIS-10 Māori data collection. Adapted with permission from ref. [38]. 2014 NZMA.

Our extensive efforts since the inception and development of POIS have ensured a study that enables us to obtain meaningful and appropriate Māori specific findings [37]. POIS-10 Māori design and analyses are explicitly underpinned by key Māori models of health and well-being, specifically the Meihana Model [38], and Te Puawaitanga o Ngā Whānau (six markers of whānau flourishing [33]). These provide positive holistic overviews of the multiple, fundamental and interconnected components required to achieve positive health outcomes for Māori. The Meihana Model has been chosen due to its utility in a range of health and research settings, as well as its focus on both the patient (i.e., injured person in our case) and whānau. The imagery of a waka hourua (a traditional Māori double-hulled canoe) allows the mapping and exploration of key characteristics (depicted as aku; crossbeams) in our study’s quantitative and qualitative components, including the explicit relationships between each of these and the injured person and whānau (depicted as hiwi; hulls) (Figure 2). Aligning with the multiple components of the Meihana Model [38], our study will capture the wide-ranging impacts of injury, including individual-level to community-level, 12 years post-SIE (Figure 2). Many factors and aspects of the multi-item measures used can be mapped to multiple components of the Meihana Model. For instance, we have mapped life satisfaction to the wairua (spirituality) component but it also closely linked to the whānau component. 

### 1.3. POIS-10 Māori Aims and Objectives

Using Māori models of health and well-being [33,35,38], POIS-10 Māori aims to understand and identify the factors contributing to long-term (12 years post-SIE; 10 years since last POIS interview) experiences and outcomes (positive and negative), for injured Māori and their whānau. The specific objectives of POIS-10 Māori are to:

1. Quantitatively describe significant life events, employment variations, comorbidities, new injury events and injury-related health service utilisation over the past 10 years (i.e., since the last interview at 24 months post-SIE);
2. Quantitatively investigate 12-year post-SIE outcomes, as informed by the Meihana Model [38];
3. Quantitatively determine which characteristics (including baseline socio-demographic and health-related, SIE-related and post-SIE-related) predict outcomes at 12 years post-SIE;
4. Qualitatively explore long-term experiences of, and barriers and facilitators of access to, health and rehabilitation services, ACC and whānau flourishing, for injured Māori and their whānau;
5. Work with key advisors and organisations to meaningfully interpret findings and identify appropriate opportunities for future interventions to improve experiences and outcomes for injured Māori and their whānau.

## 2. Experimental Design

A prospective cohort study extension following participants (via quantitative and qualitative interviews) to 12 years post-injury, 10 years since the last POIS interview [20,21]. POIS interview data will be linked to electronic data from ACC and the NMDS. Figure 1 provides an overview of the study.

## 3. Procedure

### 3.1. Participants

POIS-10 Māori participants will be Māori POIS participants previously recruited after a SIE via ACC’s entitlement claims register between 2007–2009, and who participated in the first POIS interview at 3 months post-SIE (*n* = 566; Figure 1). Participants were aged 18–64 years inclusive, and lived in one of five regions of New Zealand (Auckland, Manukau City, Gisborne, Otago and Southland) at the time of their SIE. Of the 566 Māori participants, 405 completed the 12-month post-SIE interview (71% follow-up) and 384 completed the 24-month post-SIE interview (68% follow-up). We will attempt to contact all Māori POIS participants who did not decline long-term follow-up (96% agreed to long-term follow-up; *n* = 544). Due to our strong POIS follow-up rates, engaged participants, access to multiple contact details, and by increasing our tracing efforts for POIS-10 Māori, we estimate a 65% follow-up (~*n* = 350).

### 3.2. Quantitative Data 

#### 3.2.1. POIS-10 Interviews at 12-Year Follow up

We will collect in-depth data from participants about a range of characteristics and outcomes that have occurred over the 10 years since the last POIS data collection (at 24 months post-SIE). We will contact participants using multiple contact details they previously provided for themselves and alternative contacts (e.g., a whānau member or friend). For those unable to be contacted via these routes, we will use updated contact details provided by ACC. Interview data will be collected from participants via a structured interviewer-administrated telephone survey (~1 h). These extensive interviews will also include several free text response options throughout to ensure the capture of additional rich information from participants. Interviews will be administered via a secure web-based management system, REDCap (Research Electronic Data Capture) [39]. Participants who are unable to complete a telephone interview will be offered a hard copy of the questionnaire via postal mail or a kanohi-ki-te-kanohi (face-to-face) interview. 

Validated predictors and outcome measures, consistent with previous POIS data collection, will be used to maximise the longitudinal nature of our study. As depicted in the Meihana Model [38] (Figure 2), the data will comprise Injured person-level factors including the 8-item Flourishing Scale [40]; World Health Organization Disability Assessment Scale (WHODAS) [41], and the ability to speak and understand everyday te reo Māori. Whānau and support network factors include whānau flourishing measures used in POIS [21,28,42], household income [43], and participation in unpaid activities [44]. Tinana (physical health/functioning) measures include HRQoL (EQ-5D) [45,46,47], alcohol and drug use (AUDIT-C) [48], physical activity [49], and chronic conditions (21 items). Hinengaro (psychological and emotional well-being) measures include screening for minor psychiatric disorders (General Health Questionnaire-12) [50], and depression and anxiety (Kessler-6) [51]. Wairua (connectedness and spirituality) factors include life satisfaction, relationship satisfaction [21,28,42], and comfort and strength in faith and spiritual beliefs (FACIT-Sp) [28,52]. We will also measure aspects of the Taiao (physical environment) of the injured person and whānau (e.g., living arrangements, participation in paid work, employment tenure, job satisfaction, strain and turnover, and physical and mental work-related assertion) [23,44,53,54]. We will also collect information about other key factors, including participants’ experiences of racism [55], and major life events (using the Social Readjustment Rating Scale) [56], that have occurred both within the past 12 months and over the past 10 years, to evaluate the cumulative impact of a wide range of common stressors (e.g., divorce, death of a family member).

#### 3.2.2. E-Data between 24-Months and 12-Year Follow up

Complementing the other Meihana Model components, we will also investigate Iwi Katoa (the availability of services and systems of support) for injury participants and their whānau. Administrative data relating to injury-related hospitalisations occurring across the 10 years since the POIS 24-month interview will be received from the NMDS. We will also receive administrative data from ACC about new subsequent injuries occurring over the past 10 years (e.g., funded health services, ACC support and claims processes, and earnings-related compensation), and ongoing claim entitlements from the original injury 12 years ago. 

### 3.3. Qualitative Data

**Figure 3:**
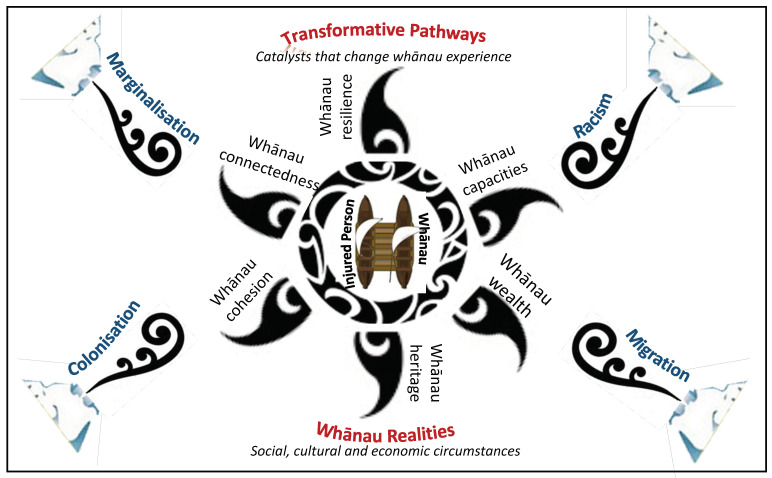
Qualitative component underpinned by Meihana and Te Puawaitanga o Ngā Whānau models [33,38].

We will conduct in-depth qualitative kanohi-ki-te-kanohi interviews with 15–20 purposively selected POIS-10 Māori participants (with a range of age, sex, geographic region, hospitalisation, long-term ACC support and injury outcomes) and their whānau, if they consent to participate. To ensure a diversity of participants, we will invite participants for this qualitative component after all quantitative interviews have been conducted. Participants and whānau will be asked, in more detail than quantitative interviews allow, about significant life events since their last POIS interview, how these and their injuries have affected the multiple components of the Meihana Model [38], (including wider components captured by Ngā Hau e Whā; the four winds) and Te Puawaitanga o Ngā Whānau [33], for flourishing whānau. We will also explore how participants’ experiences relating to the SIE have influenced subsequent healthcare, rehabilitation and ACC engagements (positive and negative). Exploring how Māori experience life after events such as injury, and how these impact on long-term well-being and the ability for injured Māori and their whānau to flourish [33,57] is of particular interest. Interview guides will be developed using the Meihana Model [38] and Te Puawaitanga o Ngā Whānau [33] (Figure 3) to gain detailed insights into dynamic relationships between injury, the dimensions of hauora [38], and whānau flourishing [33] (Figure 2 and Figure 3). Understanding these factors in relation to long-term post-injury outcomes and recovery, will aid in further identifying factors critical to flourishing [33,57]. Interviews (undertaken by VN) will be 1–1.5hr long, conducted at a venue preferred by participants, and (with consent) will be audio-recorded and transcribed.

### 3.4. Ethical Approval

POIS-10 Māori has received ethical approval from the Health and Disability Ethics Committees New Zealand (MEC/07/07/093/AM07).

## 4. Expected Results

### Analyses

To conduct quantitative analyses, POIS-10 interview data, ACC and NMDS e-data will be linked using unique person-level de-identifiers and event ID numbers, and summary variables of interest will be derived. We will also combine POIS data with that obtained in POIS-10 Māori to develop regression models (i.e., generalised linear models for continuous outcomes, modified Poisson regression with robust standard errors for binary outcomes) [58], for each key outcome at 12 years post-SIE. Models will be adjusted to allow for repeated measures (i.e., generalised estimated equation models, generalised linear mixed models) [59]. 

To produce stable estimates of coefficients in regression-based models, at least 10 cases are required for each parameter estimated. Therefore, assuming 10% have a particular binary outcome, our likely POIS-10 Māori cohort (~*n* = 350) will be sufficient to estimate 3–4 parameters in a single model; more parameters will be possible when the outcome is continuous. We are aware that loss-to-follow-up is a concern with longitudinal studies; we will have a dedicated focus on ensuring as many POIS-10 Māori participants as possible are able to be reached, but, as we have done before, we will consider multiple imputations or other techniques such as inverse probability weighting to address missingness and conduct sensitivity analyses, as appropriate [25]. 

To address objectives (Obj) 1 and 2, we will calculate the burden (e.g., frequency, nights in hospital) of injury-related hospitalisations and of injury claims (e.g., frequency, weekly compensation, service provision, claim type) over the same 10-year period. Informed by the Meihana Model [38], we will estimate (with 95% Confidence Intervals) prevalence, incidence and changes over time for key characteristics and outcomes using the linked datasets. These estimates will inform the building of Poisson regression models in Obj 3.

To address Obj 3, we will develop regression models to examine the direct effects of the socio-demographic, health and SIE-related predictors on the outcomes 12 years post-SIE, after adjustment for a wide range of confounders. Decisions about the inclusion (or not) of predictors and potential confounders will be informed by existing literature, previous POIS analyses to 24 months post-SIE, and Objs 1 and 2 findings. ACC e-data will be used to identify when participants exit the ACC scheme; 12-year outcomes will be compared between those who exited early, later or have not-yet-exited. Key outcome trajectories over time (i.e., recovery pathways) will be described as we have done previously [45], although we have not done this specifically for Māori before.

To address Obj 4, we will conduct an interpretive thematic analysis, thoroughly analysing interview transcripts and identifying themes [60,61]. The Meihana Model [38] and Te Puawaitanga o Ngā Whānau [33] (Figure 3) will also be used to develop frameworks to guide analyses. Consistent with qualitative methods used in previous kaupapa Māori research projects [28,37,62,63,64], the qualitative research team will discuss and collaboratively interpret these data and emerging themes [65]. Interviews will be transcribed and NVivo software will be used to manage the qualitative data. NVivo allows for consistent coding schemes and provides tools to query and audit the coding processes. In doing so, more robust interpretations of data can be achieved [66].

To address Obj 5, our advisory group, established for earlier Māori injury outcomes research projects, with some additions for POIS-10 Māori, will continue to guide the project direction, collaboratively interpret findings and advise on quantitative questionnaire development, analyses of primary interest and relevance for Māori, and significant aspects to explore in qualitative interviews and analyses. Our advisors have diverse expertise in Māori health research, delivery and rehabilitation service provision and use, including ACC. Their wealth of expertise from across the health and rehabilitation sector will also assist in identifying appropriate opportunities for future interventions that can improve outcomes and experiences for injured Māori and their whānau.

## 5. Discussion

Adopting a mixed-methods approach, POIS-10 Māori provides a unique opportunity to explore positive and negative long-term post-injury outcomes for Māori in New Zealand, and identify the social, financial, health and psychological factors that impact these outcomes. POIS-10 Māori will also explore the determinants of whānau flourishing and the barriers Māori face when accessing ACC and healthcare services. Expanding on the original POIS study [20], POIS-10 Māori will enable key points in the complex injury and rehabilitation pathway to be identified providing foci for future interventions to improve injured Māori and whānau outcomes, and the support required for Māori and their whānau to flourish after injury.

Our findings from POIS-10 Māori will also inform ACC’s strategic focus on wider well-being, its impact on recovery from injury and the potential impact on an ageing population. Our findings will shed light on the ongoing impacts of injury on disability, health and well-being, participation in work and unpaid activities. Functional status 24 months post-injury [25] will continue to inform ACC discussions related to outcome measurement timing across the injury recovery pathway, which outcomes are important to measure, and what happens to injured people once ACC entitlements cease. Overall, this study aims to improve Māori and whānau outcomes and experiences post-injury, resulting in health gains for Māori, whānau and wider Māori communities.

## Data Availability

Not applicable for this protocol paper.
